# NPM1 is a Novel Therapeutic Target and Prognostic Biomarker for Ewing Sarcoma

**DOI:** 10.3389/fgene.2021.771253

**Published:** 2021-11-26

**Authors:** Yangfan Zhou, Yuan Fang, Junjie Zhou, Yulian Liu, Shusheng Wu, Bin Xu

**Affiliations:** ^1^ The First Affiliated Hospital of Anhui Medical University, Hefei, China; ^2^ The First Affiliated Hospital of (University of Science and Technology of China) USTC, Hefei, China

**Keywords:** NPM1, tumor microenvironment, immunotherapy, Ewing sarcoma, ESC348884

## Abstract

Ewing sarcoma (ES) is a cancer that may originate from stem mesenchymal or neural crest cells and is highly prevalent in children and adolescents. In recent years, targeted therapies against immune-related genes have shown good efficacy in a variety of cancers. However, effective targets for immunotherapy in ES are yet to be developed. In our study, we first identified the immune-associated differential hub gene NPM1 by bioinformatics methods as a differentially expressed gene, and then validated it using real time-PCR and western blotting, and found that this gene is not only closely related to the immune infiltration in ES, but also can affect the proliferation and apoptosis of ES cells, and is closely related to the survival of patients. The results of our bioinformatic analysis showed that NPM1 can be a hub gene in ES and an immunotherapeutic target to reactivate immune infiltration in patients with ES. In addition, treatment with NPM1 promoted apoptosis and inhibited the proliferation of ES cells. The NPM1 inhibitor NSC348884 can induce apoptosis of ES cells in a dose-dependent manner and is expected to be a potential therapeutic agent for ES.

## Introduction

Ewing sarcoma (ES) is a highly aggressive sarcoma of the bone and soft tissue, and is the second most prevalent bone tumor in the world (Gaspar et al., 2015). Currently, surgery combined with radiotherapy remains the main treatment modality for ES, and little progress has been made in the treatment of ES in the last three decades (Gorlick et al., 2013). A previous study have shown that ES occurs primarily as a site-specific fusion between a member of the erythroblast transformation-specific (ETS) family of transcription factors and the EWSR1 gene (Jo, 2020). However, the mechanisms underlying ES progression and metastasis are unknown. Therefore, there is a need to develop new therapeutic targets for the management of ES.

One option is the use of immuno-oncology which is attracting increasing interest, and immunotherapy has achieved good results in cancers treatment such as pancreatic and lung cancers (Steven et al., 2016; Morrison et al., 2018). An increasing number of immune targets has been developed, and the development of immune checkpoint inhibitors such as PD-L1, PD-1, and CTLA-4 as drugs has shown good results (Chong et al., 2021). However, in ES, monoclonal antibodies against PD-1 or PD-L1 have not shown significant clinical efficacy (Morales et al., 2020). Therefore, comprehensive analysis of the relationship between immune-related genes and patients with ES and the development of new immunotherapeutic targets may provide a new reference for the treatment and prognostic assessment of patients with ES.

With the rapid development of bioinformatics technology, many tools for identifying biomarkers have been developed (Zarabi et al., 2020), among which the weighted gene co-expression network analysis (WGCNA) and single-sample Gene Set Enrichment Analysis (ssGSEA) algorithms have been applied to the screening of a large number of tumor biomarkers (Tian et al., 2020; Zhou et al., 2021). In our study, we aimed to use bioinformatics to identify and test new therapeutic targets for ES.

## Materials and Methods

### Data Acquisition

A working flow chart is depicted in Supplementary Figure S1, We downloaded the dataset of ES from the Gene Expression Omnibus (GEO) database (https://www.ncbi.nlm.nih.gov/geo/). The dataset GSE34620 (Postel-Vinay et al., 2012) contains the RNA sequences of 117 patients with ES; GSE17674 (Savola et al., 2011) contains the RNA sequences of 18 normal skeletal muscle samples, and RNA sequence and survival information of 44 patients with ES. In addition, GES45544 (Agelopoulos et al., 2015) and sarcoma data from The Cancer Genome Atlas (TCGA) were used for the validation of the final results. All relevant information of these 3 GEO datasets was showed in Supplementary Table S1.

### Bioinformatic Analysis of the Immune Microenvironment in Patients With ES

We obtained the relevant gene sets of 28 immune cell species from the literature (Jia et al., 2018), then we used the R package “GSVA” (Hänzelmann et al., 2013) to score immune cells in 117 patients. Based on the immune cell scoring, we divided the patients into three clusters using unsupervised clustering and used the R package “pheatmap” to draw an immune scoring heat map to visualize the differences in immune infiltration among the three groups. Based on the heat map, we selected the two groups with the greatest difference in immune infiltration and classified them into high and low immune infiltration groups; we screened the two groups for differential genes [false discovery ratio (FDR) < 0.05, |logFC|>1], and a differential gene heat map was drawn.

We used the R package “ESTIMATE,” which is an algorithm developed by Yoshihara et al., for sample immune scoring, stromal scoring, assessing tumor purity and estimated scoring for 117 patients for the next step of WGCNA analysis (Yoshihara et al., 2013).

### Selection of Soft Thresholds and the Construction of Immune-Related Modular Trait Relationships in Patients

WGCNA is a bioinformatics algorithm developed by Langfelder and Horvath (2008), which is used to cluster highly related genes into modules according to the phenotype of interest. The connectivity between genes needs to meet the criteria of a scale-free network. In a scale-free network, the logarithm [log(k)] of the number of nodes containing connectivity k and the logarithm {log [p(k)]} of the probability of occurrence of the node should show a negative correlation, and the correlation coefficient between them should be greater than 0.85. This coefficient is called the soft threshold, and higher the soft threshold, higher is the chance of conforming to the scale-free network rules. Individual modules were then identified by hierarchical clustering and dynamic branching cuts, with a unique color assigned to each module as an identifier. Gene significance (GS) and module affiliation (MM) values were then calculated to associate modules with immune-related traits. The corresponding module gene information was extracted for further analysis.

### Selection of Immune-Related Differential Genes

Using the dataset GSE17674, we screened for differentially expressed genes between normal skeletal muscle tissue and ES tissue (FDR < 0.05, |logFC|> 2). The data of the intersection of differential genes with immune traits were taken and a Venn diagram showing immune-related differential genes was plotted.

### Selection of Hub Genes

We used the string online website (https://string-db.org) to construct the immune-related differential gene protein interaction network, and then used the “cytohubba” plugin in “Cytoscape” software to select the top five most correlated hub genes using the Matthews correlation coefficient (MCC) algorithm.

### Cell Culture

ES is a cancer that may originate from stem mesenchymal or neural crest cells (Kersting et al., 2018), and according to the literature, this experiment used RD-ES and A673 cell lines purchased from the American Type Cell Culture (ATCC) as disease group and mesenchymal stem cells (MSCs) purchased from Cyagen (Guangzhou, China) as normal control group (Li et al., 2019). We used 89% Dulbecco’s Modified Eagle Medium (DMEM; Gibco, United States), 10% fetal bovine serum (Gibco, United States), 1% double antibody (100 U/ml penicillin and 100 mg/ml streptomycin), complete medium, and 25T culture flasks to culture MSCs, RD-ES, and A673 cells at 37°C and 5% CO_2_, respectively.

### Real Time-PCR

We used Trizol (Sigma, United States) to extract total RNA from the cells, which was reverse transcribed into cDNA using a reverse transcription kit (Takara, Japan). Real-time PCR was performed using SYBR Premix Ex Taq (Takara, Japan) according to the manufacturer’s instructions. PCR reaction conditions: denaturation at 95°C for 10 s, annealing at 60°C for 15 s, and extension at 72°C for 30 s. This cycle is amplified for 45 times, and the melting curve is analyzed after the cycle. We design primers by using the online website “primerBank” (pga.mgh.harvard.edu). The primer sequences are listed in Supplementary Table S2.

### Western Blotting

The protein lysis solution was prepared using radioimmunoprecipitation (RIPA) buffer, phosphatase inhibitor, and phenyl methane sulfonyl fluoride (PMSF) at 97:2:1. After lysis for 30 min, loading buffer was added, and the mixture was boiled at 100°C for 10 min. Proteins were resolved by electrophoresis on 12% sodium dodecyl sulfate–polyacrylamide gel electrophoresis (SDS-PAGE). After electrophoresis and membrane transfer, the membranes were blocked with 5% skimmed milk for 2 h at room temperature. The membranes were then incubated with NPM1 antibody (1:2000, Abcam, United Kingdom) and β-actin antibody (1:2000, Abcam, United Kingdom) overnight at 4°C and washed three times with Tris-buffered-saline-Tween 20 (TBST) for 10 min. Finally, the polyvinylidene fluoride (PVDF) membranes were incubated with secondary antibodies (Sigma, United States) at room temperature for 1 h and fluorescence was detected using a western blot analysis system with electrochemiluminescence (ECL) fluorescent agent. In natural PAGE gel experiments, samples were not denatured by heating, and electrophoresis was performed in the absence of SDS.

### Cell Viability Assays

RD-ES cells (approximately 4 × 10^3^ cells) and A673 cells (approximately 1 × 10^4^ cells) were seeded in 96-well plates at a plate laying time of 24 h. The cells were stimulated with the NPM1 inhibitor NSC348884 at concentrations of 0, 0.5, 1, 1.5, 2, and 3 µM. The inhibitor ESC348884 was dissolved in dimethyl sulfoxide (DMSO) (Sigma, United States). Four replicate wells were used for each concentration. After 24 h of incubation, 10 µl of Cell Counting Kit (CCK)-8 reagent was added to each well and incubated for another 2 h. The absorbance was measured at 450 nm. The cell survival rate was calculated as follows: Average OD value of dosed cells/average OD value of control cells = survival rate.

### Apoptosis Assay

RD-ES cells and A673 cells in the logarithmic growth stage were inoculated in 6-well plates at approximately 1 × 10^6^ cells per well; after the cell confluence reached 70%, stimulation was carried out with a gradient of inhibitor concentrations at 0, 0.5, 1, 1.5, 2, and 2.5 µM. MSCs were stimulated with 0, 1, 2, and 3 µm NSC348884. After 24 h, cells were collected by trypsin digestion, washed three times with 1 × phosphate buffer solution (PBS), and the number of cells was adjusted to approximately 1 × 10^6^. The apoptosis rate was detected using an Annexin V-fluorescein isothiocyanate (FITC)/propidium iodide (PI) double-stained apoptosis detection kit (Bestbio, Shanghai, China) and flow cytometry (BD Biosciences, Franklin Lakes, New Jersey, United States).

### Evaluation of the Effectiveness of Immunotherapy

To evaluate the effect of NPM1 expression on immunotherapy in patients with ES, we calculated Tumor Immune Dysfunction and Exclusion (TIDE) scores of 117 patients with ES from GSE34620 using the TIDE website developed by Harvard University (http://tide.dfci.harvard.edu/). Based on the median expression of NPM1, the samples were divided into high- and low-expression groups, and the differences in TIDE scores between the two groups were compared.

### Statistical Methods

Bioinformatic analyses were implemented using R 4.0.3, and the Wilcoxon rank sum test was used for the analysis between two groups. The Bayesian test was used for the selection of differential genes. External experiments were repeated three times, and statistical analyses were performed using GraphPad Prism 6.0 (GraphPad Software), and Student’s t-test was used for comparison between the two groups.

## Results

### Immune Infiltrative Subtypes of ES

**FIGURE 1:**
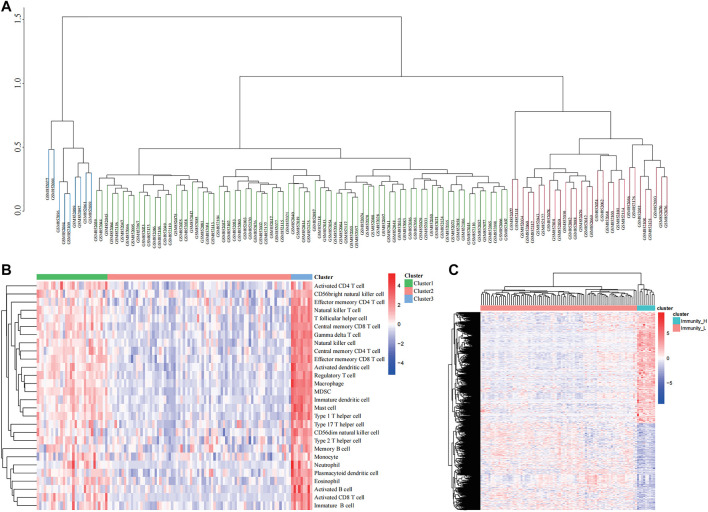
Clustering based on immune microenvironment in Ewing sarcoma (ES). **(A)** The samples were divided into three groups based on single-sample Gene Set Enrichment Analysis (ssGSEA) immune scoring. **(B)** The enrichment levels of 28 immune-related cells in the high immune cell infiltration group (Immunity_H), middle immune cell infiltration group (Immunity_M), and the low immune cell infiltration group (Immunity_L). **(C)** Heatmap showing the difference between Immunity_H and Immunity_L.

As per the ssGSEA algorithm, we scored each ES sample for the enrichment of 29 immune cells, and the 117 patients with ES were then divided into three groups: cluster1, cluster2, and cluster3 using an unsupervised clustering method, and a clustering tree was drawn (cutoff = 1, Figure 1A). In addition, the enrichment scoring heat map also visualizes the difference in degree of immune infiltration among the three groups, where cluster1 is the moderate immune infiltration group with 30 samples, cluster2 is the low immune infiltration group with 78 samples, and cluster3 is the high immune infiltration group with 9 samples (Figure 1B). Using the R package “limma,” we screened immune-related genes for differences between the high and low immune groups and found that 3,342 genes were differentially expressed, and the differences between the two groups were shown in a heat map (Figure 1C).

### WGCNA Selection of Immune-Related Genes

**FIGURE 2:**
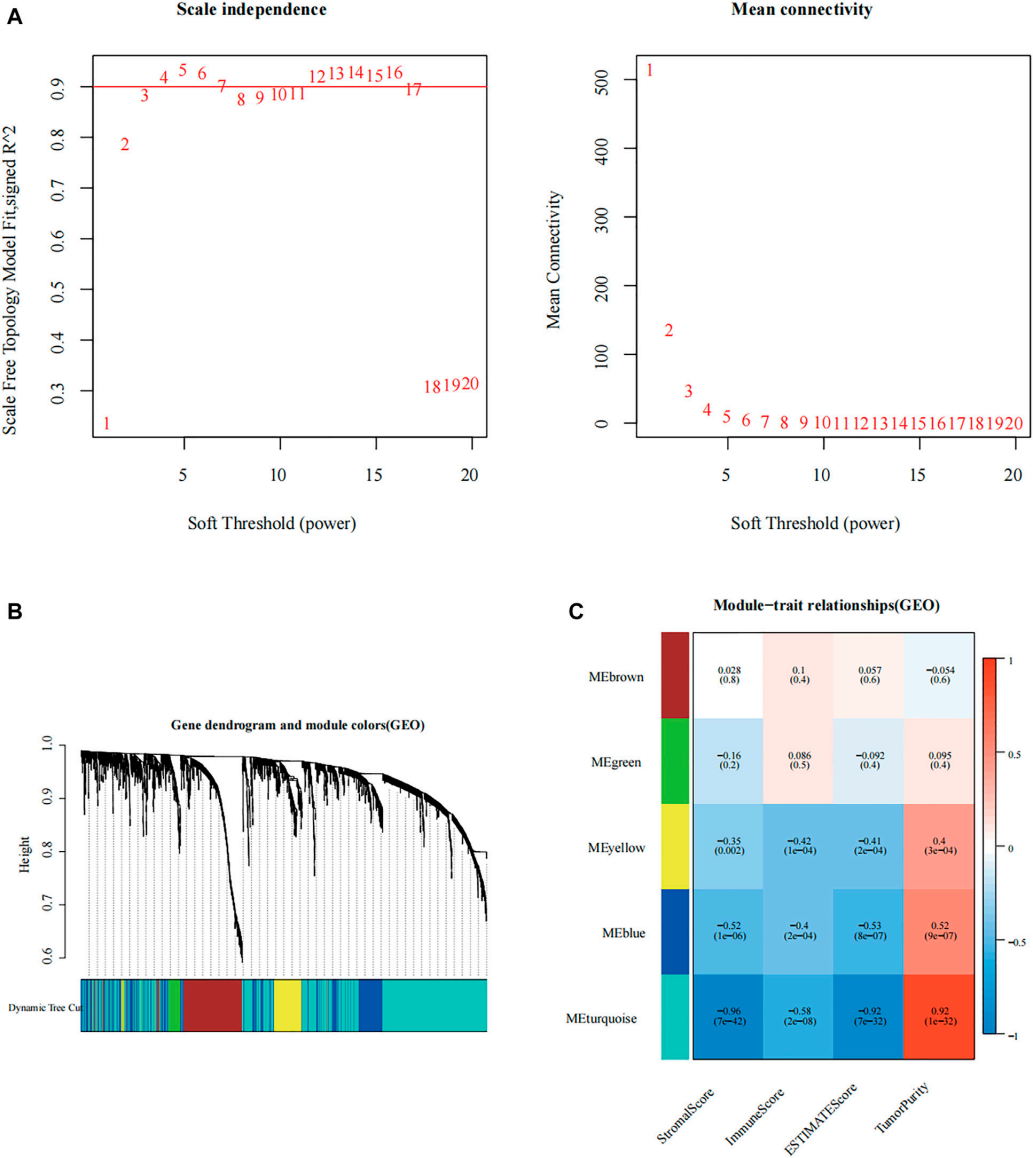
Construction of immune-related modular by weighted gene co-expression network analysis (WGCNA). **(A)** Analysis of the scale-free fit index of the 1–20 soft threshold power (*β*), and the average connectivity of 1–20 soft threshold power. **(B)** Genes are grouped into various modules by hierarchical clustering, and different colors represent different modules. **(C)** Relationship between the gene modules and the immune-related information.

We first calculated the soft threshold power *β* and propose its co-expression similarity to calculate the adjacency relation. We used the function “ickSoftThreshold” to perform network topology analysis *via* the R package “WGCNA.” In the subsequent analysis, the soft threshold power *β* was set to 3, as the scale independence reached 0.85, and had relatively high average connectivity (Figure 2A). Based on *β* = 3, the 3,342 immune-related differential genes were grouped into five modules, including brown, green, yellow, blue, and turquoise (Figure 2B). Combined with the immune score, stromal score, estimate score, and tumor purity, the module correlation heat map was drawn (Figure 2C). According to previous studies, lower levels of immune infiltration are often associated with a poorer prognosis in patients with tumor (Zhou et al., 2021). Therefore, we selected the turquoise module with the strongest negative correlation with immune scoring for inclusion in the follow-up study.

### Enrichment Analysis of Turquoise Module-Related Genes

**FIGURE 3:**
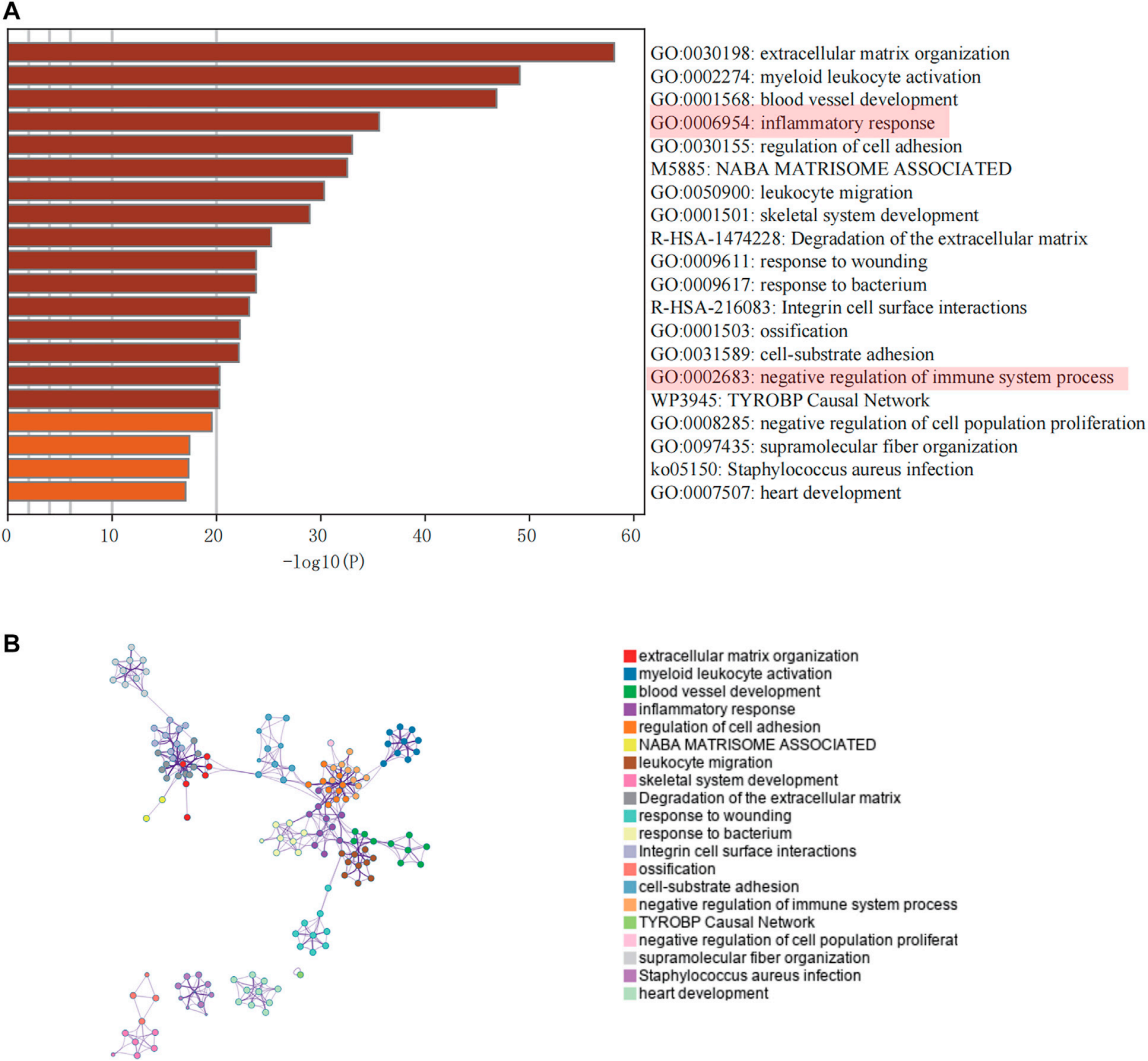
Enrichment analysis of core modules. **(A)** The top 20 enriched terms are shown in a bar chart. **(B)** Network diagram showing the association between each enrichment item.

Genes included in the turquoise module were analyzed using the web tool “Matascape” for pathway and process enrichment analysis. Several biological functions related to immunity were discovered (Figures 3A,B). The negative correlation between our screening module and immune infiltration was again verified by the “negative regulation of the immune system process.”

### The Selection of Hub Genes

**FIGURE 4:**
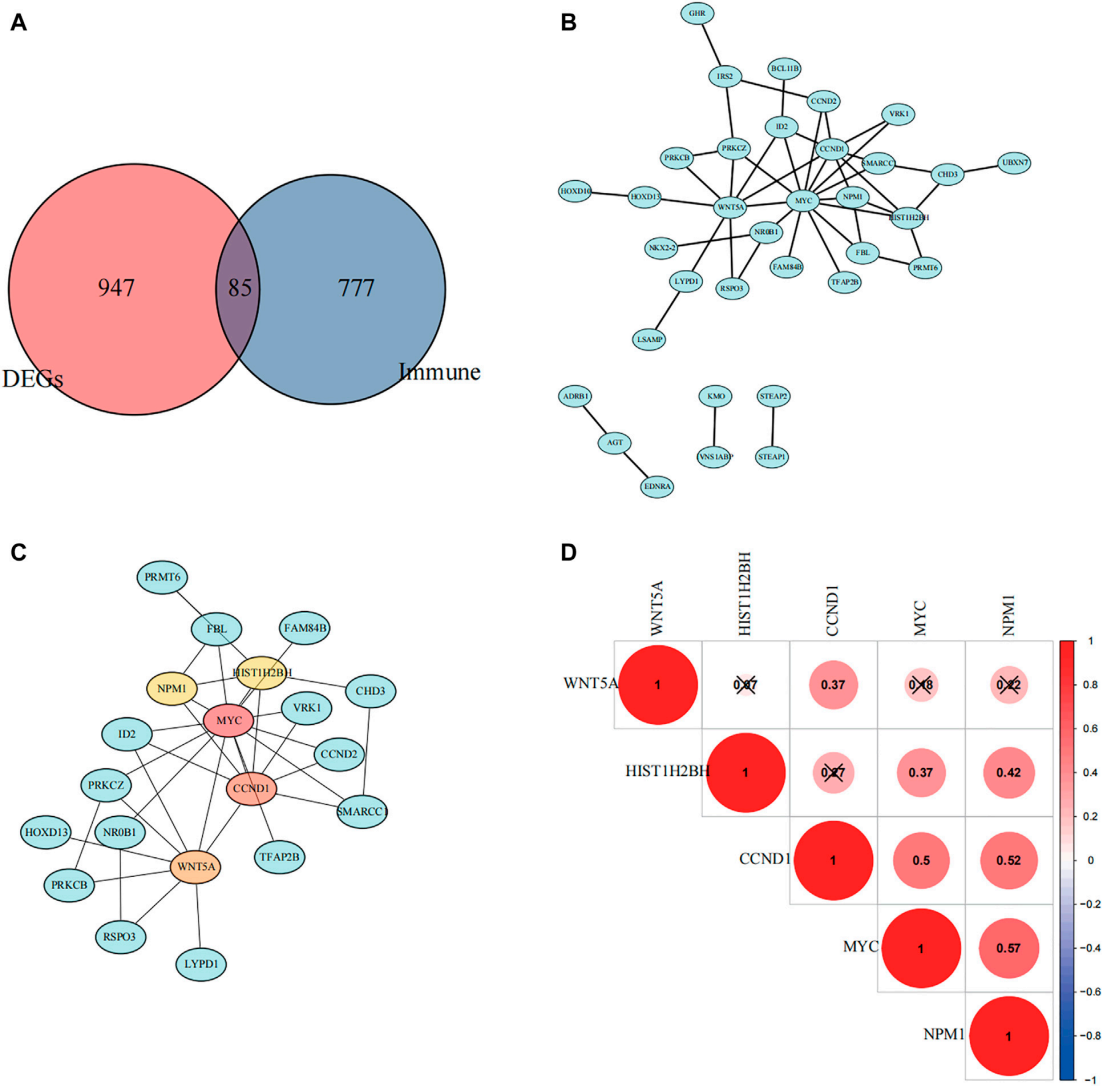
Selection of hub genes. **(A)** A Venn diagram showing immune-related differential genes. **(B)** Protein interaction network mapping based on negatively associated differential immune genes. **(C)** Top five differential genes were obtained according to the Matthews correlation coefficient (MCC) algorithm. **(D)** The correlation between the five hub genes was calculated based on the Pearson correlation coefficient, and red color indicates positive correlation.

**FIGURE 5:**
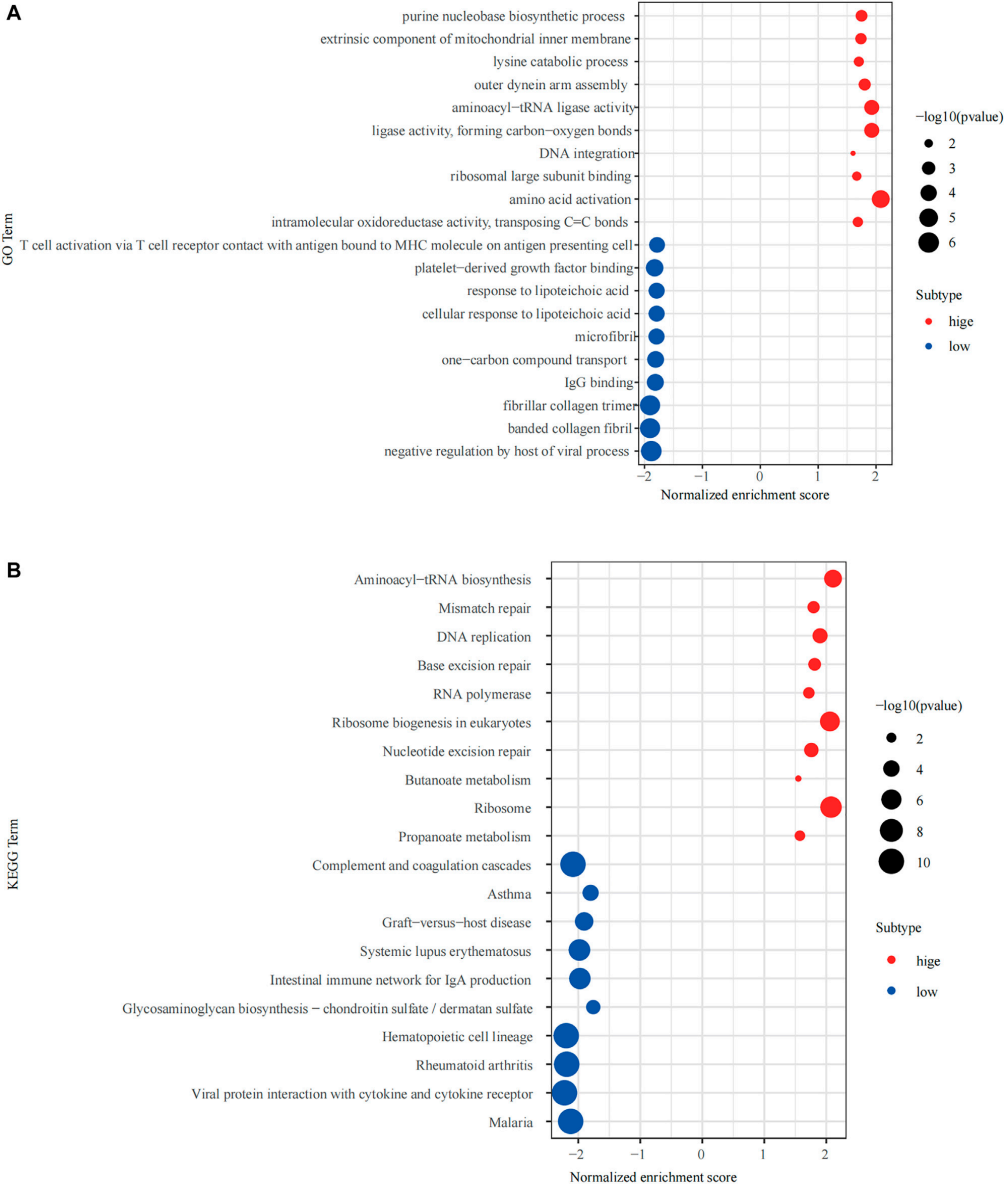
Gene Set Enrichment Analysis (GSEA) of the high and low expressing NPM1 groups. **(A)** The results of Gene Ontology (GO) enrichment analysis. **(B)** The results of Kyoto Encyclopedia of Genes and Genomes (KEGG) enrichment analysis.

From GSE17674, we obtained 1,032 differentially expressed genes (DEGs) between normal and ES groups (| logFC | > 2 and FDR < 0.05, Supplementary Figure S1), In addition, we excluded the genes with GS > 0 in the turquoise module, and the remaining 862 genes negatively associated with immune infiltration were selected to intersect with the differential genes to obtain 85 differential genes that were negatively associated with immune infiltration (Figure 5A). The protein interaction network (Figure 4B) was constructed using the String (https://string-db.org) online tool, optimized by “cytoscape” software, and the top 5 hub genes were obtained by “cytohubba” plugin using the “MCC” algorithm, namely, MYC, CCND1, WNT5A, NPM1, and HIST1H2BH (Figures 4C,D). By reviewing the literature, we finally selected NPM1, a poorly studied gene in ES, for inclusion in the follow-up study.

### GSEA Analysis

The R package “clusterprofiler” was used to perform Gene Ontology (GO) and Kyoto Encyclopedia of Genes and Genomes (KEGG) enrichment analysis of the high and low expression groups (Figures 5A,B). We found that multiple immune-related biological functions and pathways were enriched in the NPM1-low expression group, including “Intestinal immune network for IgA production, Viral protein interaction with cytokine and cytokine receptor, T cell activation *via* T cell receptor contact with antigen bound to MHC molecule on antigen presenting cell, IgG binding and others.”

### Relevance of NPM1 to Immunity

**FIGURE 6:**
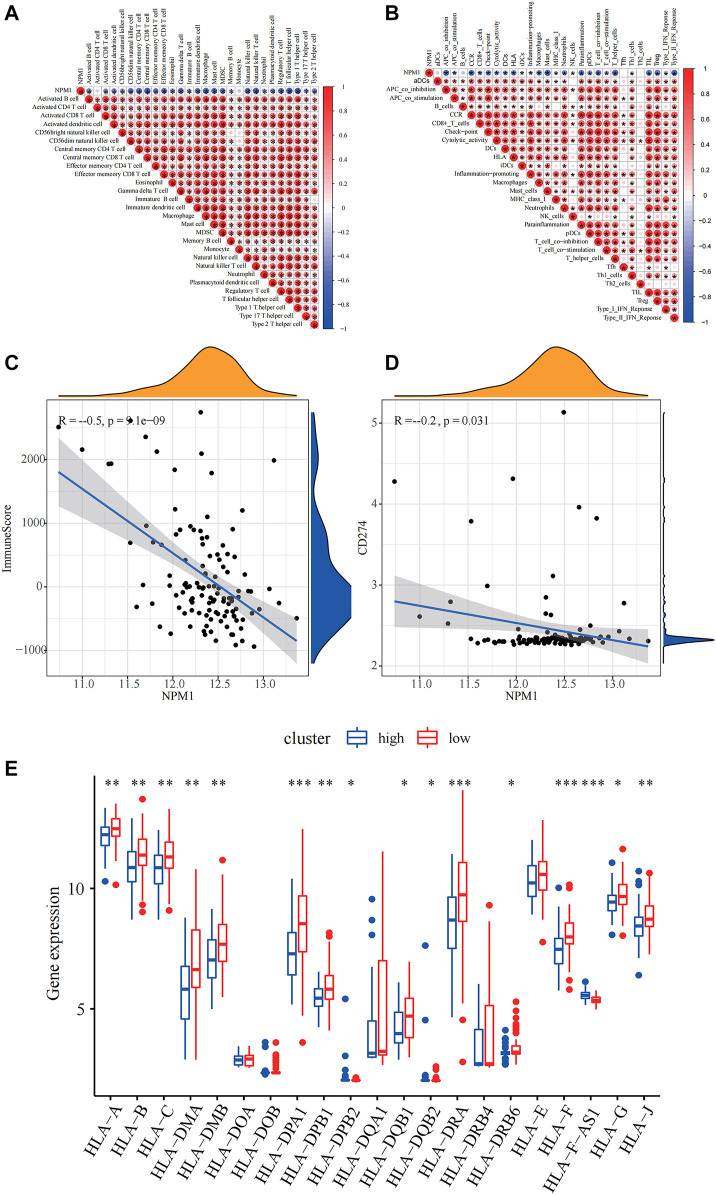
Relevance of NPM1 to immunity. **(A)** Correlation of NPM1 with single-sample Gene Set Enrichment Analysis (ssGSEA) scoring of 28 immune cells. **(B)** Correlation of NPM1 with 29 immune functions, immune-related pathways and ssGSEA scoring. **(C)** Calculation of correlation between NPM1 and immunization scoring based on Pearson correlation coefficient. **(D)** Calculation of correlation between NPM1 and PD-L1 (CD274) based on Pearson correlation coefficient. **(E)** Differences in human leukocyte antigen (HLA) family expression between high and low NPM1 subgroups are demonstrated by box plots.

Based on the correlation plot of immune cell enrichment and immune-related pathways, the enrichment of immune function, and NPM1, we found that NPM1 showed a close negative correlation with them. Moreover, the immune score and the expression of PD-L1 showed a significant negative correlation with NPM1 expression, suggesting that patients with low NPM1 expression may have better immunotherapy efficacy (Figure 6).

### Validation of the Hub Gene by RT-PCR and Western Blotting

**FIGURE 7:**
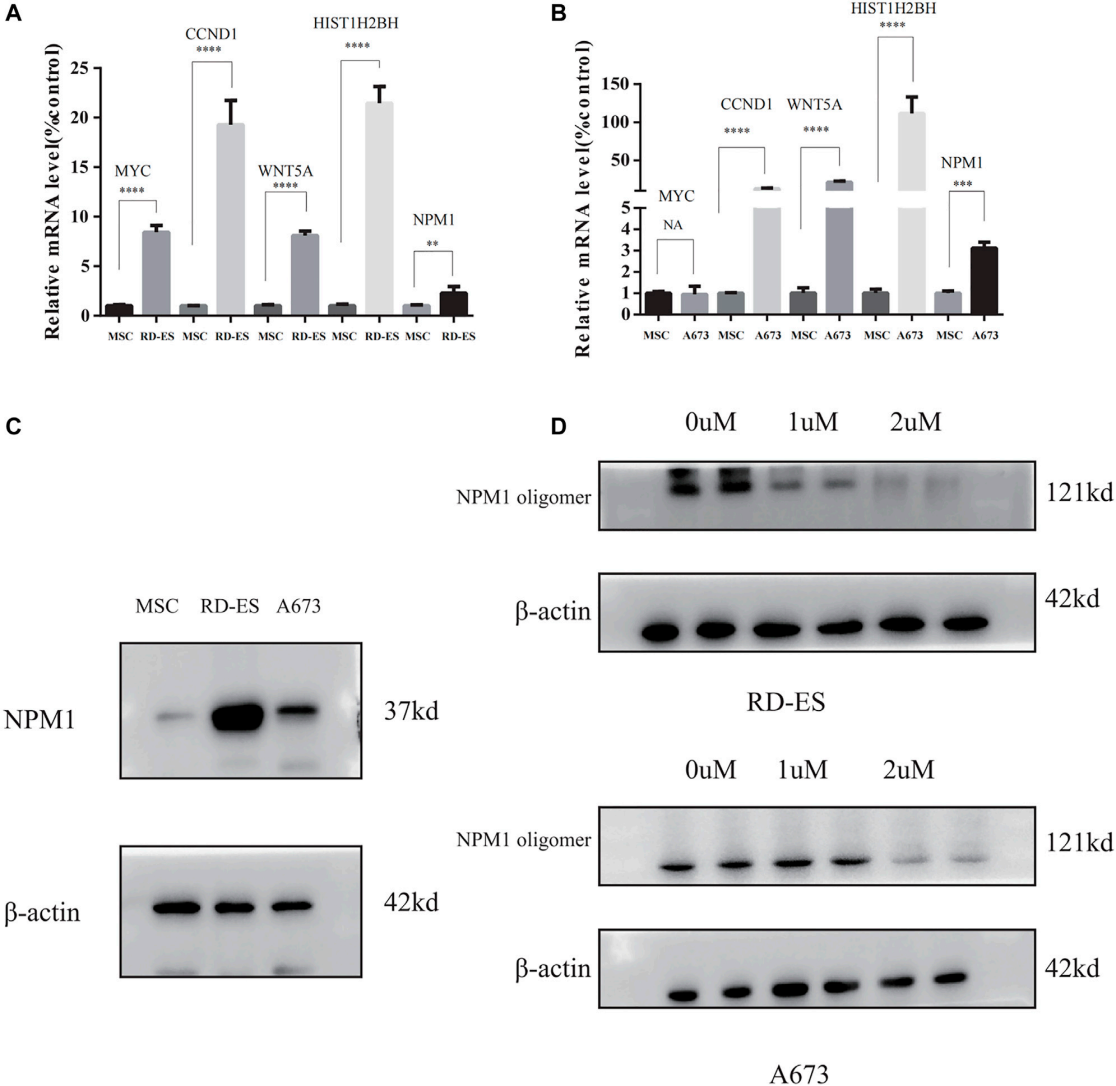
**(A,B)** The mRNA expression of MYC, CCND1, WNT5A, HIST1H2BH, and NPM1 detected between mesenchymal stem cells (MSC), and Ewing sarcoma (ES) cell lines. **(C)** NPM1 protein expression levels were higher in RD-ES and A693 cells than in MSCs by western blotting. **(D)** RD-ES and A673 cells were treated with NSC348884 at 0, 1, 2 µM for 24 h and the results of western blotting showed that NSC348884 inhibits the formation of NPM1 protein oligomers (NA, *p* > 0.05; *, *p* < 0.05; **, *p* < 0.01; ***, *p* < 0.001; ****, *p* < 0.0001).

The results of RT-PCR showed that the relative expression levels of five hub genes, including WYC, CCND1, WNT5A, HIST1H2BH, and NPM1 were higher in RD-ES cells than in MSCs (Figure 7A), while in the A673 cell line, in addition to MYC, the remaining four hub genes were also significantly overexpressed in the tumor (Figure 7B). In addition, we explored the difference in the expression of NPM1 in tumor group versus control group at the protein level. The results of western blotting showed that NPM1 was more highly expressed in ES cell lines than in MSCs (Figure 7C).

### Exploration of the Biological Function of NPM1

**FIGURE 8:**
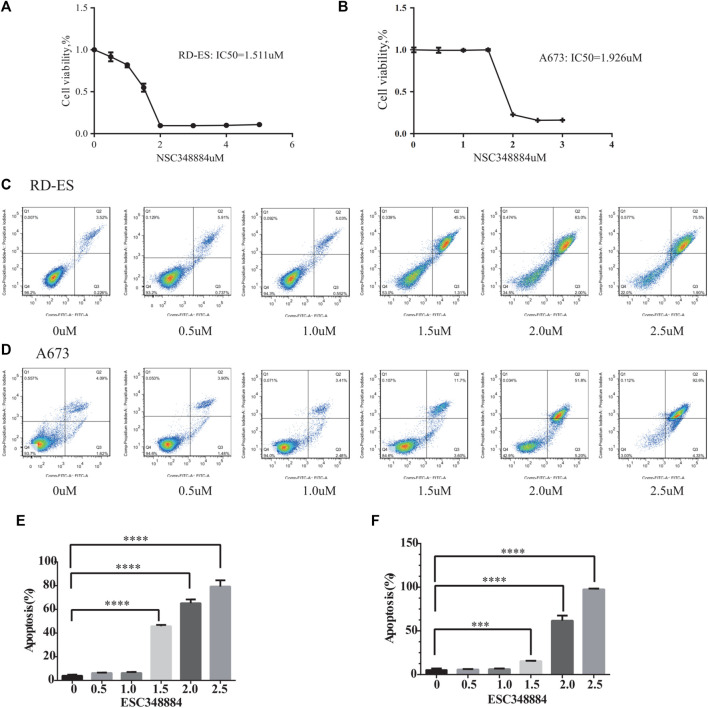
Effect of NSC348884 on the survival rate and apoptosis of RD-ES and A673 cells. **(A)** Survival rate of RD-ES stimulated by different inhibitor concentrations. **(B)** Survival rate of A673 cells stimulated by different inhibitor concentrations. **(C)** Apoptosis rate of RD-ES cells stimulated under six different concentrations, from left to right were 0, 0.5, 1, 1.5, 2, and 2.5 µM. **(D)** Apoptosis rate of A673 cells stimulated under six different concentrations of ion, from left to right were 0, 0.5, 1, 1.5, 2, and 2.5 µM. **(E)** The bar graph shows the apoptosis rate of RD-ES cells at different drug concentrations. **(F)** The bar graph shows the apoptosis rate of A673 cells at different drug concentrations (NA, *p* > 0.05; *, *p* < 0.05; **, *p* < 0.01; ***, *p* < 0.001; ****, *p* < 0.0001).

Since NPM1 may be a potential therapeutic target for patients with ES, the NPM1 inhibitor was identified by reviewing the literature as NSC348884, which can bind specifically to NPM1 and specifically interfere with the formation of NPM1 oligomers (Qi et al., 2008). In our study, we found that the synthesis of NPM1 protein oligomers was also inhibited in ES cells (Figure 7D). Therefore, we assayed cell survival by stimulating RD-ES cells and A673 with NPM1 inhibitor NSE348884 using the CCK8 kit, and plotted the concentration effect curve of NSE348884 (Figure 8B) and derived a 50% inhibition concentration (IC_50_) of 1.511 µM for this inhibitor in RD-ES cells (Figure 8A) and IC_50_ of 1.926 µM in A673 cells (Figure 8B). In ES cell lines, the apoptosis rate increased with increasing drug concentration. When NSC348884 concentrations were greater than 1.5 µM, the apoptosis rate was >45%. The apoptosis rate was greater than 50% at ESC348884 concentrations greater than 2 µM in A673 cells, and the apoptosis rate of cells increased with increasing drug concentration (Figures 8C,D,F,G). Together, these data support the notion that NSC348884 induces apoptosis in ES cells through the inhibition of NPM1. Therefore, targeted therapy against NPM1 is of great importance for the treatment of ES.

### Relationship Between NPM1 Expression and Survival Rates

**FIGURE 9:**
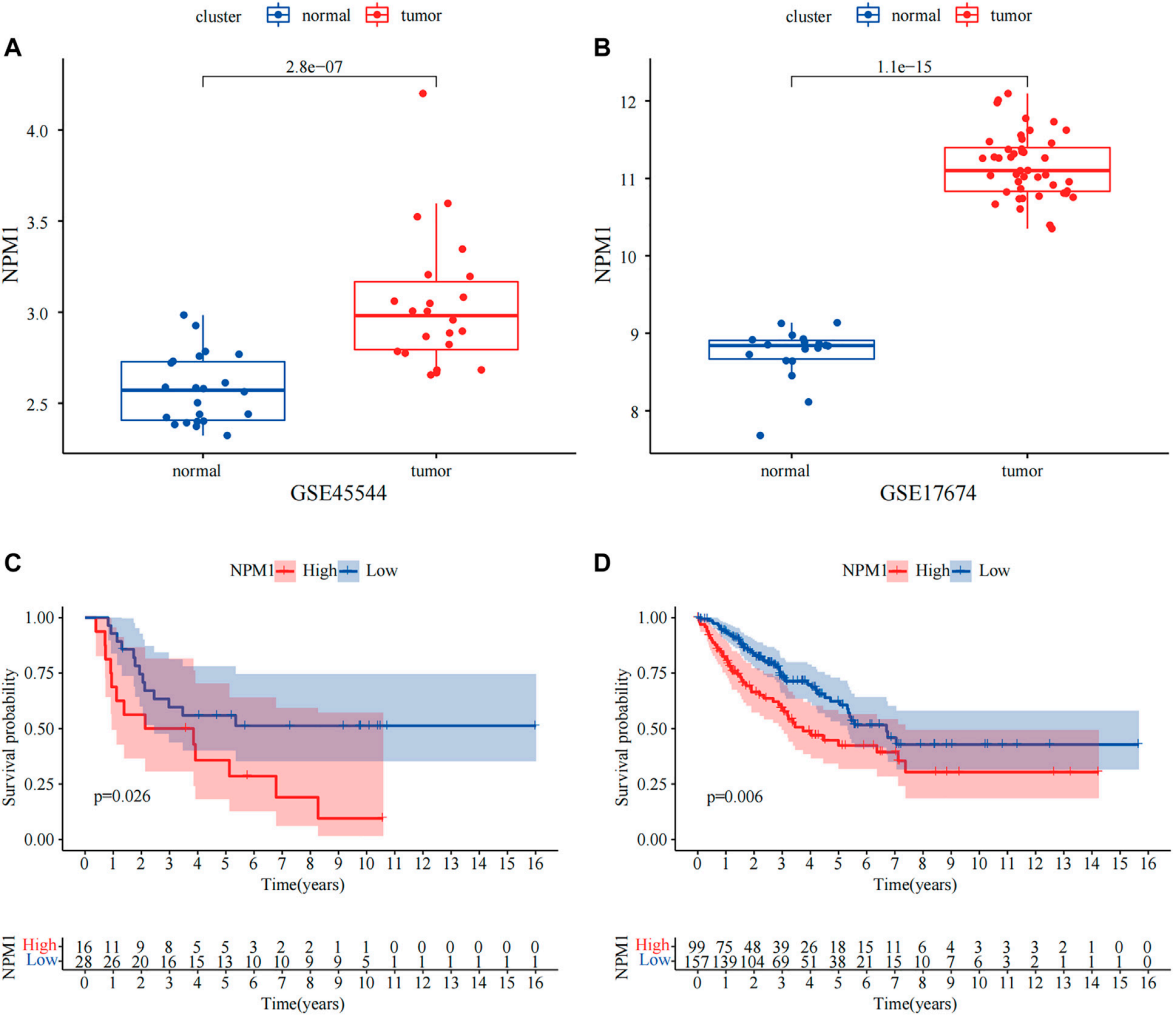
External dataset validation of the variability of NPM1 and correlation with survival. **(A,B)** In GSE17674 and GSE45544, NPM1 was highly expressed in ES groups. **(C,D)** Gene Expression Omnibus (GEO) and The Cancer Genome Atlas (TCGA) data demonstrate that high NPM1 expression is strongly associated with poor prognosis.

In both GSE17674 and GSE45544, we found that NPM1 showed significantly high expression in ES (*p* < 0.05, Figures 9A,B). Moreover, we found that NPM1 was closely associated with the survival of patients with ES, and high expression of NPM1 in the ES data of GSE17674 and the sarcoma dataset of TCGA often predicted a poor prognosis for patients (*p* < 0.05, Figures 9C,D). These results suggest that high NPM1 expression is a clear risk factor for patients with ES.

### Overview of NPM1 in Human Cancer

**FIGURE 10:**
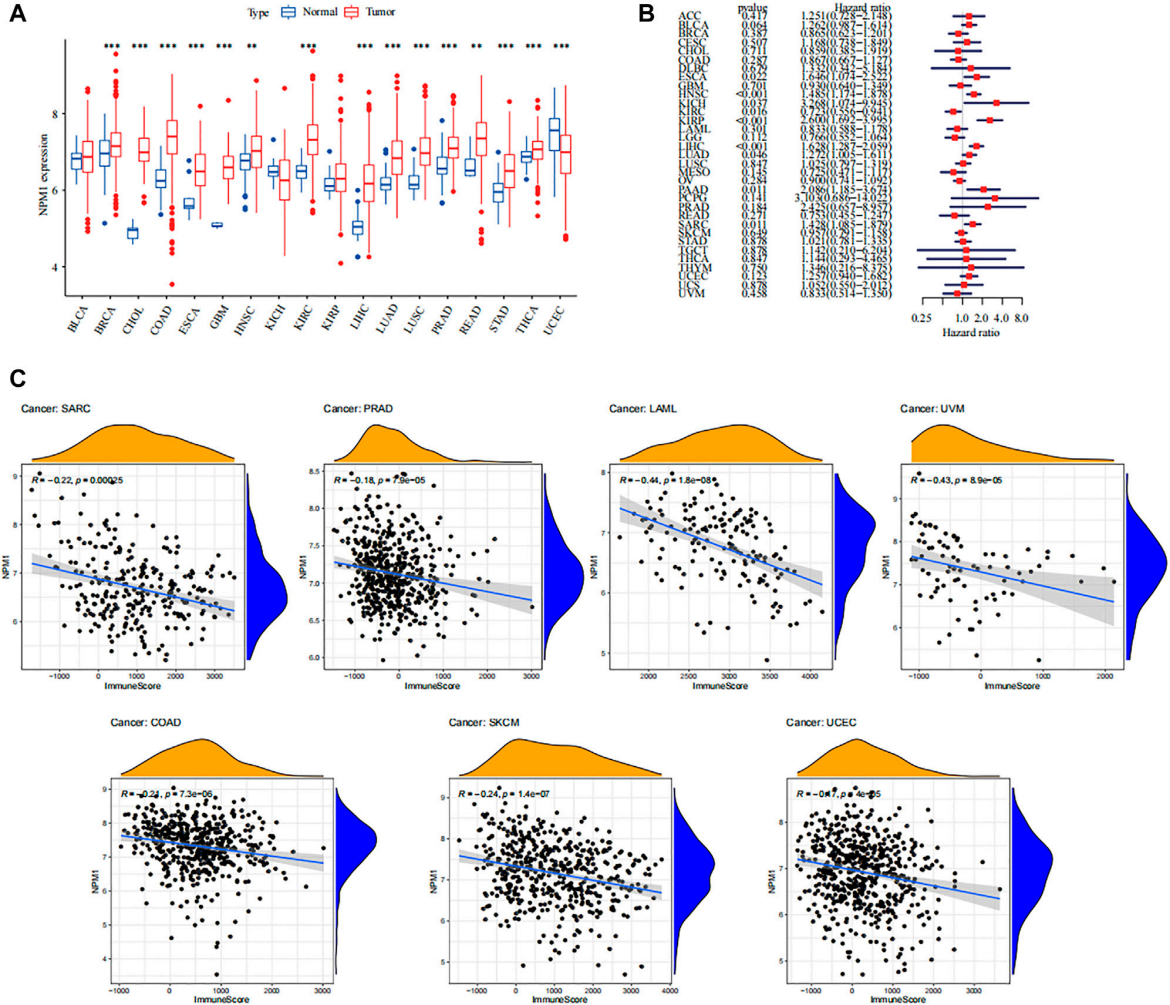
Overview of NPM1 in human cancer. **(A)** The mRNA expression of NPM1 between tumor and normal control tissues was assessed from The Cancer Genome Atlas (TCGA) database. **(B)** Univariate Cox regression analyses estimating prognostic value [overall survival (OS)] of NPM1 in different cancer types from TCGA database. **(C)** Association between immune scoring and NPM1 expression in multiple tumors.

Overall, NPM1 is overexpressed in multiple cancer types. Probably due to insufficient sample size, differential expression of NPM1 was not observed in some cancers including bladder cancer (BLCA) and head and neck squamous cell carcinoma (HNSC) (Figure 10A). Moreover, we found that NPM1 was strongly associated with patient prognosis in several cancers and acted as an unfavorable prognostic factor (Figure 10B). Furthermore, to explore the association between NPM1 and immune infiltration in other cancers, we analyzed the association between NPM1 and immune scoring in multiple cancers, and in cancers with statistically significant differences (*p* < 0.001), we found a strong negative association between NPM1 and patients’ immune scoring (Figure 10C). In particular, in sarcoma, the results in TCGA were also validated with the results of our GEO analysis. In addition, through the online website TISIDB (http://cis.hku.hk/TISIDB/), we found that NPM1 expression was negatively correlated with populations of lymphocytes, MHC molecules, immunostimulators, chemokines, and receptors in most of the 30 cancer species (Supplementary Figure S2).

### TIDE Score to Assess the Impact of the Expression of NPM1 on Immune Checkpoint Inhibitor Treatment

Previous studies have shown that high TIDE prediction scores indicate potential immune evasion, suggesting that patients are less likely to benefit from ICI therapy (Chen et al., 2021). In our results, the TIDE score of NPM1 group was lower than that of NPM1 group (*p* = 0.011). Moreover, NPM1 expression showed a significant negative correlation with TIDE score. Supplementary Figure S3 shows the significance of NPM1 in guiding immunotherapy in patients with ES.

## Discussion

Currently, the prognosis of most patients with ES is poor, even with a comprehensive treatment strategy (Gaspar et al., 2015). In addition, ES is highly invasive and metastatic, and patients often suffer from bone and lung metastases, leading to deterioration of health (Cao et al., 2015). Therefore, it is necessary to develop new therapeutic targets for ES and consider the treatment of the disease from various aspects, such as immune infiltration and proliferation inhibition. According to our analysis, NPM1 is not only highly expressed in patients with ES, but is also closely associated with poor patient prognosis and immune infiltration; therefore, the development of drugs targeting NPM1 is essential.

NPM1, also known as nuclear phosphoprotein, consists of 294 amino acids and is an abundant and multifunctional nucleolar phosphoprotein that frequently shuttles between the nucleus and the cytoplasm of cells and is involved in a variety of important biological activities (Karimi Dermani et al., 2021). Previous studies have shown that NPM1 plays an important role in cell growth and proliferation by regulating cell cycle progression and centrosome replication (Okuda, 2002). NPM1 plays an important role in both solid tumors and leukemia. The World Health Organization (WHO) classification has defined acute myeloid leukemia (AML) with NPM1 mutations as a distinct entity (Heath et al., 2017). In breast cancer, colon cancer, and other cancers, overexpression of NPM1 often results in a poor prognosis prediction for patients (Liu et al., 2012; Zeng et al., 2019).

In recent years, the successful use of ICI therapy in tumors has led to an increasing interest in immune-targeted therapies (Wang et al., 2016). Immune targets such as PD-L1, PD-1, and CTLA-4 have been shown to be effective in a variety of tumors, and targeting immune cells to activate checkpoints has been shown to be the most effective way to activate anti-tumor immune responses (Rotte, 2019). In recent years, more and more immune checkpoint inhibitors have been developed, such as PD-L2, CD80, CD86 and so on (Sugiura et al., 2019; Wennhold et al., 2021). They both achieved good results in the reactivation of immune cells. Previous studies have shown that clinically detected tumors often require immune escape to evade antitumor immune responses in order to grow; therefore, a higher degree of immune infiltration often predicts a better prognosis for patients with tumor (Gajewski et al., 2013; Yi et al., 2020). The relevance of NPM1 in immune infiltration in patients with tumors has been demonstrated in several tumors. According to Qin et al., NPM1 upregulates PD-L1 transcription and suppresses T-cell activity in triple-negative breast cancer (Qin et al., 2020). In lung adenocarcinoma, NPM1 is also involved in immune infiltration and its expression is closely related to the presence of a variety of immune cells (Liu et al., 2021). Our study also found a significant negative correlation between NPM1 and immune infiltration in patients with ES. GSEA enrichment analysis also showed that patients with low NPM1 expression were enriched for multiple immune-related biological functions and pathways. Moreover, we found that NPM1 showed a significant negative correlation with various immune cells, immune functions, and human leukocyte antigen (HLA) families. Therefore, we speculate that inhibition of NPM1 can reactivate immune infiltration in patients with ES, and NPM1 is expected to be a new immunotherapeutic target.

NSC348884, a specific inhibitor of NPM1, binds to NPM1 and specifically disrupts the hydrophobic pocket structure of the amino terminus, thereby inhibiting the formation of oligomers and disrupting their abnormal function in cancer cells (Qi et al., 2008). Our study revealed that NSC348884 also inhibited NPM1 oligomers in ES cells. Moreover, the inhibition of NPM1 function can promote the apoptosis of ES cells.

In addition to NPM1, our study identified MYC, CCND1, and WNT5A as potential therapeutic targets for ES. They may jointly influence the immune infiltration of ES and the development of tumorigenesis through interaction with NPM1. In an earlier report, Kawano et al. reported that microRNA-20b promotes cell proliferation in ES cells by increasing MYC expression (Kawano et al., 2017). A previous study showed that MYC-driven cancer cells promote tumorigenesis through immune escape, suggesting that MYC-induced tumors may be particularly sensitive to immuno-oncological intervention, which is consistent with our findings, suggesting a strong negative correlation between MYC and immune infiltration in ES. The high expression of CCND1 in ES has been demonstrated in several studies (Fagone et al., 2015; Palombo et al., 2019), and overexpression of this gene contributes to the dysregulation of the cell cycle in cancer, leading to the proliferation of tumor cells (Palombo et al., 2019). A study by Zhe Jin et al. showed that WNT5A could promote ES cell migration by upregulating CXCR4 expression. These studies again validate our results and show that NPM1, CCND1, WNT5A, and NPM1 can act as key genes for ES.

Our study has some limitations: first, the interactions of NPM1 with other proteins and its correlation with immune cells are based only on bioinformatic analysis and require experimental validation. This will be discussed in a future study. Second, NPM1 has an effect on ES cell apoptosis, but the molecular mechanism behind this is not clear, and we lacked clinical samples and clinical prognostic information to validate our results. Finally, NPM1 was identified as a hub gene based on the GEO dataset; however, the effectiveness of NPM1 as a therapeutic target and prognostic factor needs to be further validated.

In conclusion, the results of our bioinformatics analysis showed that NPM1 is essential for the proliferation of ES cells and can also act as an immunotherapeutic target to reactivate immune infiltration in patients with ES. In addition, treatment with NPM1 promoted apoptosis and inhibited the proliferation of ES cells. Therefore, NPM1 is expected to be an independent prognostic factor and a new therapeutic target for ES. The NPM1 inhibitor NSC348884 can induce apoptosis of ES cells in a dose-dependent manner without affecting the survival of normal cells. Therefore, it can be a potential therapeutic target for ES.

## Data Availability

Publicly available datasets were analyzed in this study. The names of the repository/repositories and accession number(s) can be found in the article/Supplementary Material.

## Abbreviations

ATCC, American Type Cell Culture; AML, acute myeloid leukemia; BLCA, bladder cancer; CCK, Cell Counting Kit; DEGs, differentially expressed genes; DMEM, Dulbecco’s Modified Eagle Medium; ES, Ewing sarcoma; ETS, erythroblast transformation-specific; ECL, electrochemiluminescence; FDR, false discovery ratio; FITC, fluorescein isothiocyanate; GO, Gene Ontology; GEO, Gene Expression Omnibus; GS, Gene significance; HLA, human leukocyte antigen; HNSC, head and neck squamous cell carcinoma; ICI, immune checkpoint inhibitor; KEGG, Kyoto Encyclopedia of Genes and Genomes; MM, module affiliation; MCC, Matthews correlation coefficient; PVDF, polyvinylidene fluoride; PI, propidium iodide; PMSF, phenyl methane sulfonyl fluoride; RIPA, radioimmunoprecipitation; ssGSEA, single-sample Gene Set Enrichment Analysis; SDS-PAGE, sodium dodecyl sulfate–polyacrylamide gel electrophoresis; TIDE, Tumor Immune Dysfunction and Exclusion; TCGA, The Cancer Genome Atlas; WGCNA, weighted gene co-expression network analysis.
