# Supplementary material for: NPM1 is a Novel Therapeutic Target and Prognostic Biomarker for Ewing Sarcoma
Source: Front Genet. 2021 Nov 26;12:771253. doi: 10.3389/fgene.2021.771253 (PMC8662625; doi:10.3389/fgene.2021.771253)
Supplement: Supplementary file 1 [file DataSheet1.docx]

## Supplementary Figure1

##
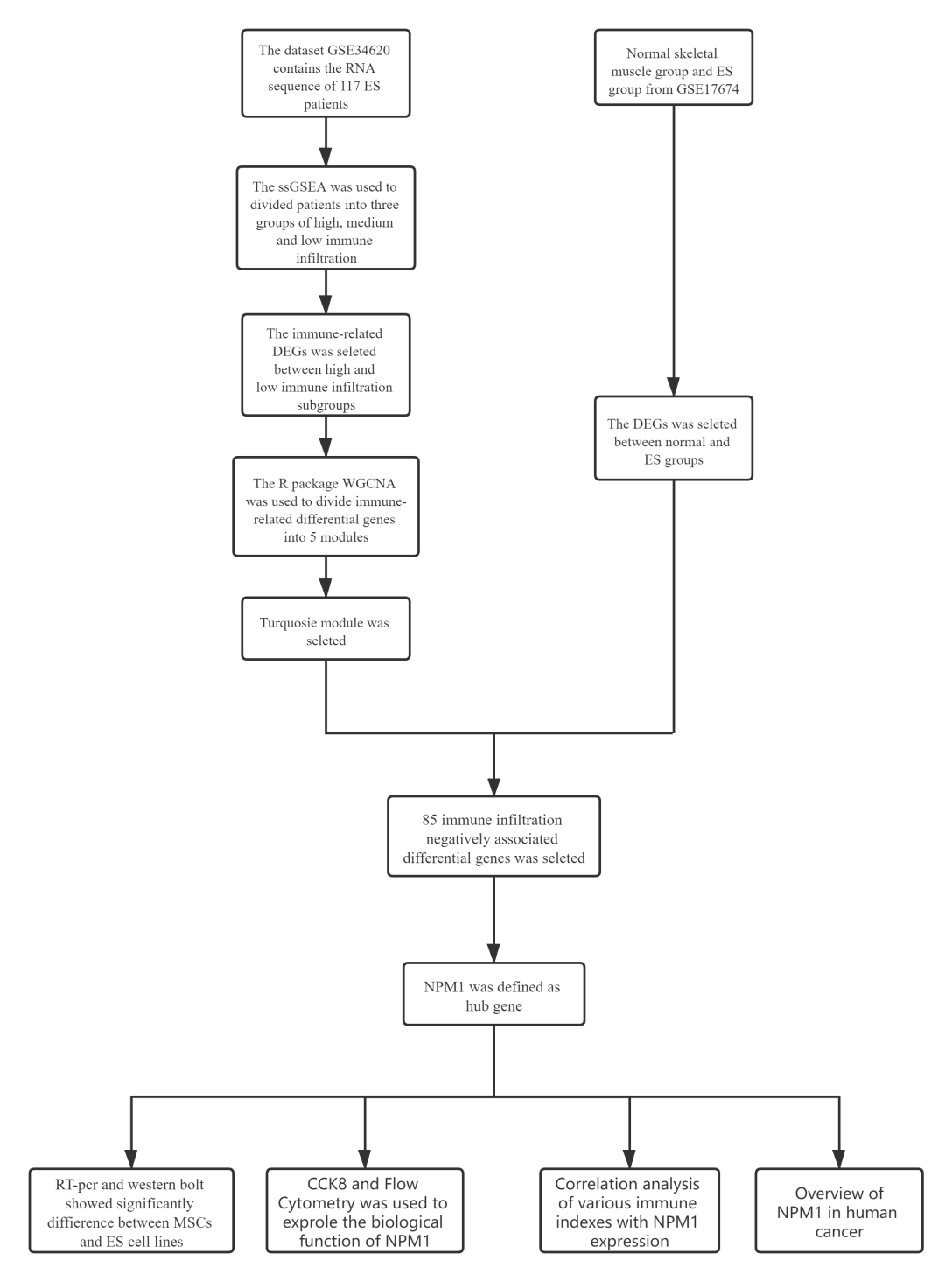


**Supplementary Figure 2. Flow chart of data collection and analysis.**

## Figure2
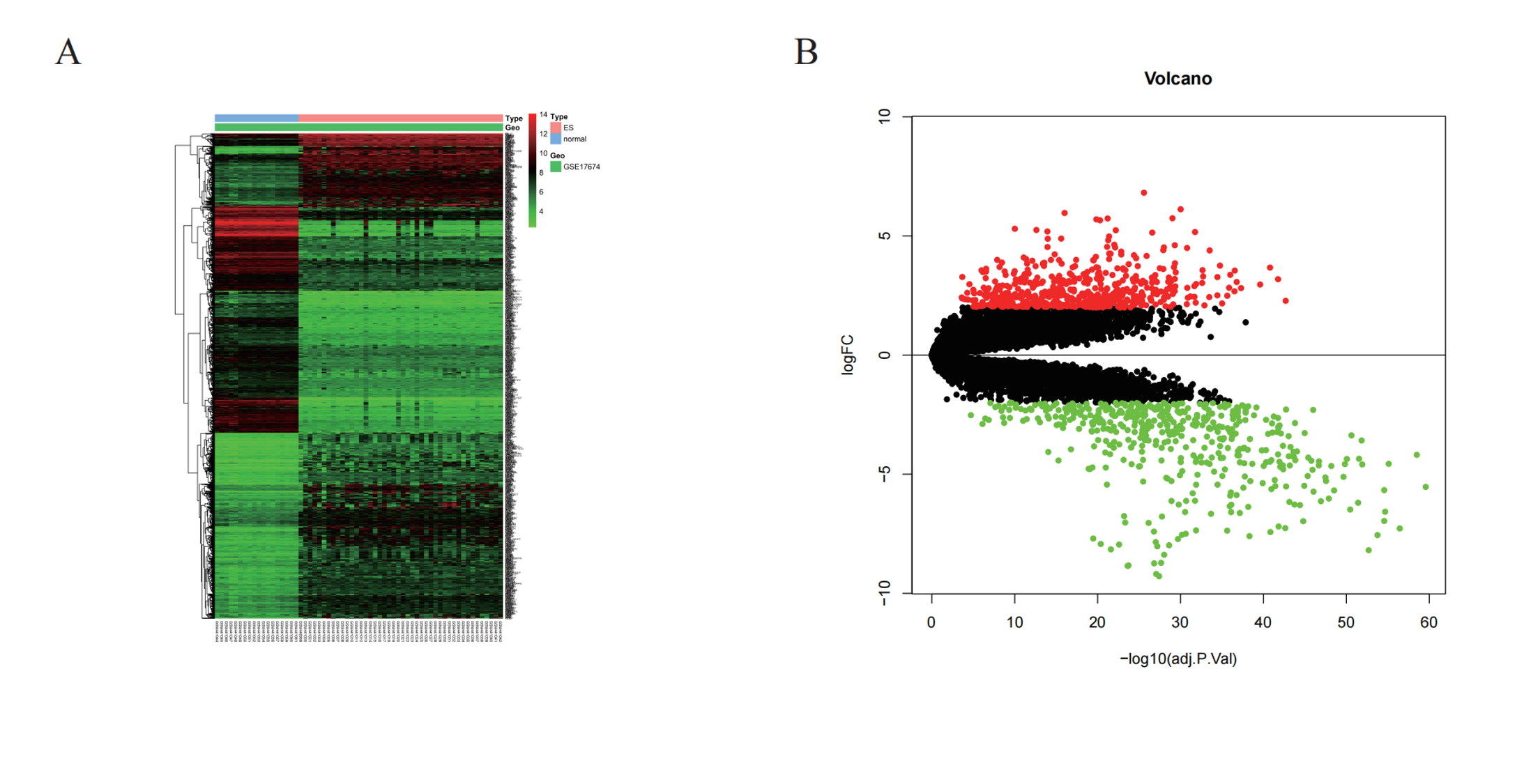


## Supplementary Figure 2. (A)Heat map of the differences between the normal and ES groups. (B) Volcano plot of the difference between normal and ES groups.


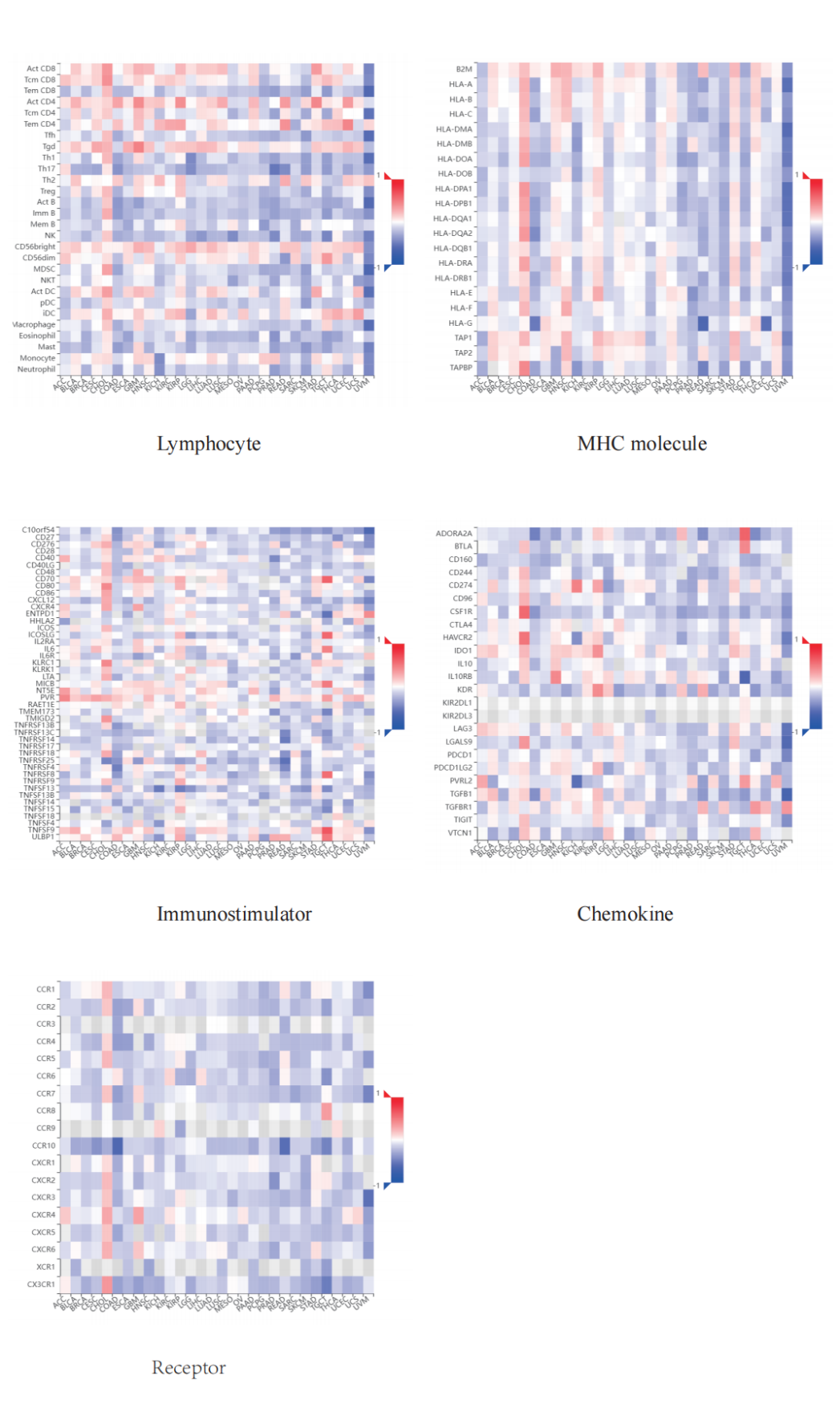


## Supplementary Figure 2. The heatmap showed the correlation between NPM1 and lymphocyte, MHC molecule, Immunostimulator, Chemokine, Receptor.

**
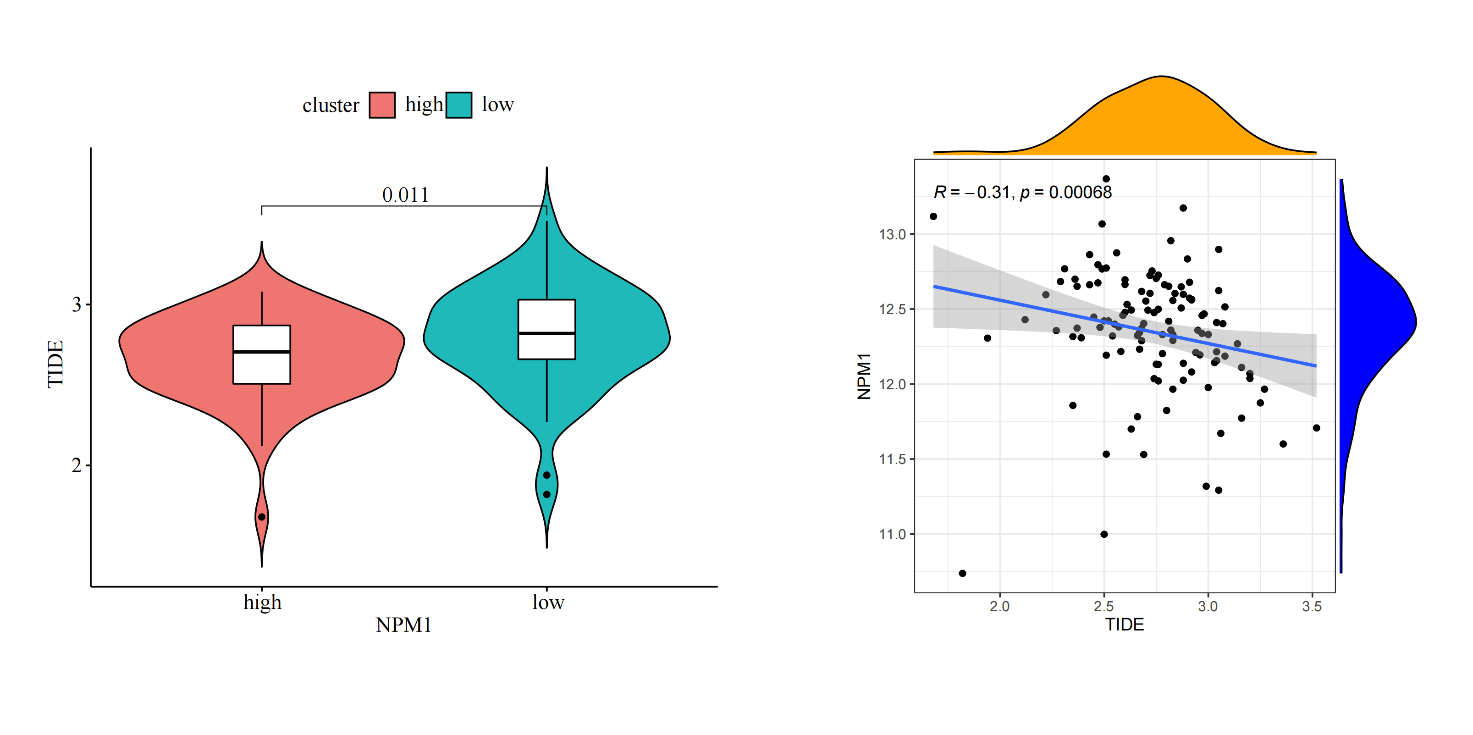
**

## **Supplementary Figure 3. TIDE score in high and low expression of NPM1 groups, and the expression of NPM1 showed significantly negative correlation with TIDE score.**

**1.4 Supplementary**

**Table1**

| **GEO accession** | **Author** | **Platform** | **Normal: ES** |
| --- | --- | --- | --- |
| GSE34620 | Postel-Vinay | GPL570[HG-U133_Plus_2] Affymetrix Human Genome U133 Plus 2.0 Array | 0-117 |
| GSE17674 | Savola | GPL570[HG-U133_Plus_2] Affymetrix Human Genome U133 Plus 2.0 Array | 18-44 |
| GSE45544 | Agelopoulos | GPL6244 [HuGene-1_0-st] Affymetrix Human Gene 1.0 ST Array [transcript (gene) version] | 22-22 |

Supplementary Table 1: Summary information of studies included in analysis

**Table2**

| MYC-Forward: GGCTCCTGGCAAAAGGTCA | MYC-Reverse: CTGCGTAGTTGTGCTGATGT |
| --- | --- |
| CCND1-Forward: GCTGCGAAGTGGAAACCATC | CCND1-Reverse: CCTCCTTCTGCACACATTTGAA |
| WNT5A-Forward: ATTCTTGGTGGTCGCTAGGTA | WNT5A-Reverse: CGCCTTCTCCGATGTACTGC |
| HIST1H2BH-Forward: CCTGGCGCATTACAACAAGC | HIST1H2BH-Reverse: CTTTGGGTTTGAACAGGCGT |
| NPM1-Forward: GGAGGTGGTAGCAAGGTTCC | NPM1-Reverse: TTCACTGGCGCTTTTTCTTCA |
| GAPDH-Forward: GGGAGCCAAAAGGGTCAT | GAPDH-Reverse: GAGTCCTTCCACGATACCAA |

Supplementary Table 2: Sequence of primers used to amplify target genes.
